# Stroke-Like Migraine Attacks After Radiation Therapy Syndrome

**DOI:** 10.31486/toj.19.0090

**Published:** 2020

**Authors:** Daniel April, Neil Lall, Andrew Steven

**Affiliations:** ^1^ Department of Radiology, Ochsner Clinic Foundation, New Orleans, LA; ^2^ Department of Radiology, Emory University School of Medicine, Atlanta, GA

## INTRODUCTION

Stroke-like migraine attacks after radiation therapy (SMART) syndrome is a delayed complication of brain irradiation.^1^ Symptoms are varied but typically consist of recurrent migraines with subsequent stroke-like symptoms and seizures.^2-5^ The associated imaging findings are unusual but can be quite characteristic on magnetic resonance imaging (MRI) and include unilateral increased T2 signal with associated gyral thickening and enhancement in the temporal, parietal, and occipital lobes.^1^ The pathogenesis is not as well understood as with other complications of radiation, such as leukoencephalopathy and radiation necrosis, but familiarity with this rare clinicoradiologic syndrome is important. We describe the case of a patient with a typical clinical presentation and imaging findings of SMART syndrome.

## HISTORY AND CASE REPORT

A 58-year-old female presented to the emergency department (ED) with a 2-day history of severe headache, right-sided hemiparesis, involuntary eye blinking, slurred speech, and confusion. Her medical history included hypertension, hyperlipidemia, type 2 diabetes mellitus, and transient ischemic attacks. Additionally, she had a history of stage IV breast cancer with an isolated left frontal lobe brain metastasis. She had undergone surgical excision and brain irradiation therapy 8 years prior to presentation. The patient had visited the ED several times during the prior 3 years with syncopal episodes and confusion. Computed tomography (CT) and MRI of the brain performed at these visits demonstrated stable postoperative and radiation changes in the left frontal lobe at the site of the patient's known metastatic disease. Two electroencephalograms (EEGs) performed at these visits demonstrated generalized slowing in the left frontal lobe without epileptiform activity. These episodes were treated as absence seizures, and she was prescribed levetiracetam (Keppra) 1 g twice daily for management.

**Figure 1.**
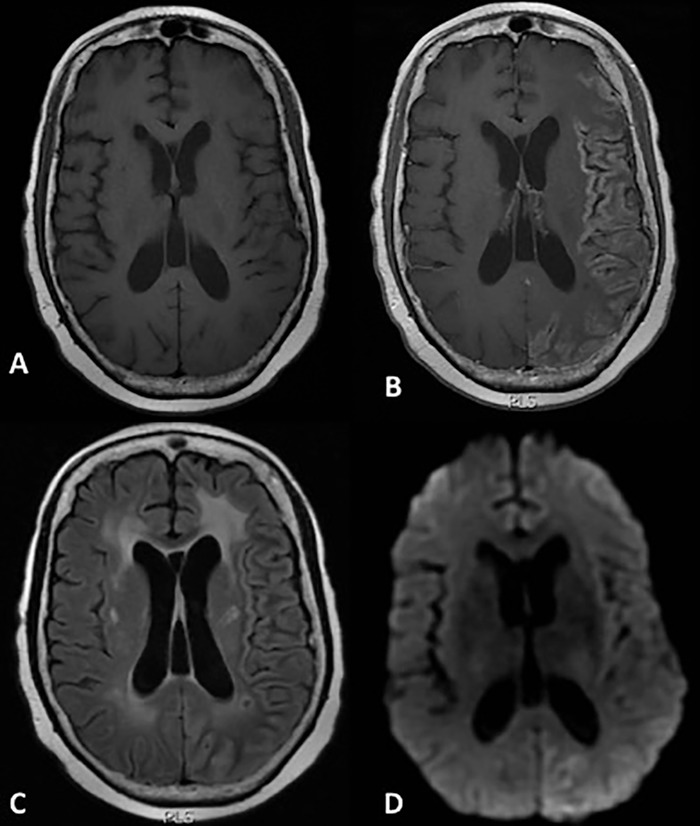
**Magnetic resonance imaging on hospitalization day 9. Axial T1-weighted images through the level of the cerebral hemispheres before (A) and after (B) the administration of gadolinium contrast demonstrate diffuse gyral enhancement throughout much of the left cerebral hemisphere with some sparing of the frontal lobe, with corresponding mildly hyperintense signal on a T2/fluid attenuated inversion recovery image (C) and diffusion restriction on diffusion weighted imaging (D).**

At this most recent ED visit, initial CT and MRI of the brain revealed stable posttreatment changes, as well as mild cerebral atrophy and chronic ischemic microvascular changes, but no acute abnormalities. The patient was admitted to the hospital, and initial complete blood count (CBC), blood culture, and paraneoplastic panel were negative. Initial lumbar puncture (LP) showed elevated white blood cells, and meningitis was suspected. On day 1 of admission, empiric antiviral treatment and antibiotics were started: intravenous acyclovir 640 mg 3 times daily for 9 days, fluconazole 400 mg once daily for 5 days, and piperacillin-tazobactam 4.5 g once daily for 3 days. Because of the concern for seizure, 1,000 mg of levetiracetam twice daily was initiated. Initial EEG revealed bilateral hemispheric slowing without focal epileptiform activity. Cerebrospinal fluid (CSF) cultures showed no growth, and repeat LP on hospital day 6 was negative. The patient had continued episodes of confusion, and because of concern for persistent seizure activity, on hospital day 7, her levetiracetam dose was increased to 1,500 mg twice daily, and lacosamide 100 mg was added on hospital day 8. On hospital day 9, the patient had a witnessed seizure with eye blinking and right-sided facial twitching that was stopped with lorazepam. Repeat MRI exhibited new mild cortical expansion and enhancement, with increased signal on T2-weighted images and diffusion restriction involving the posterior left cerebral hemisphere (Figure 1). Her lacosamide dose was increased to 200 mg twice daily, and she was transferred to the intensive care unit for further monitoring.

**Figure 2.**
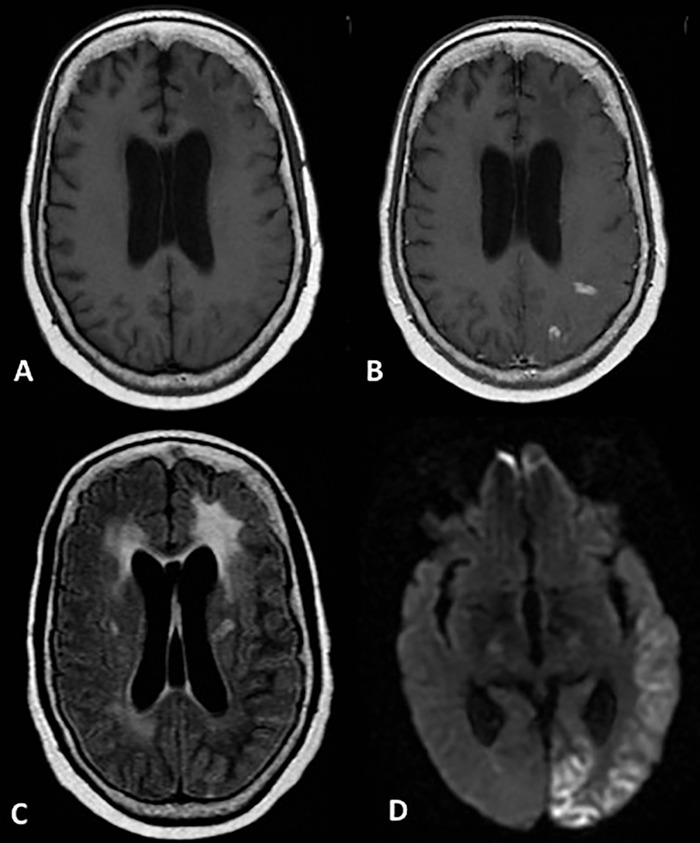
**Follow-up magnetic resonance imaging 1 week later. Axial T1-weighted images through the level of the cerebral hemispheres before (A) and after (B) the administration of gadolinium contrast demonstrate few remaining foci of cortical enhancement involving the left posterior parietal and occipital lobes, with corresponding mild residual T2/fluid attenuated inversion recovery signal along the left occipital lobe (C) and persistent cortical restricted diffusion involving the left posterior parietal and occipital lobes on diffusion weighted imaging (D).**

On hospital day 11, the patient was found unresponsive; she was intubated and transferred for a higher level of care and continuous EEG monitoring. She was extubated 13 days later, and repeat MRI showed persistent but decreased enhancement with slightly decreased hyperintensity in the posterior left cerebral cortex and persistence of diffusion restriction (Figure 2). Given the imaging appearance, evolution of imaging findings, and the patient's clinical history, a diagnosis of SMART syndrome was suspected.

The patient continued to improve slowly, but she had persistent altered mentation, aphasia, and difficulty performing activities of daily living and required percutaneous endoscopic gastrostomy (PEG) tube placement. On hospital day 20, she was switched to and maintained on levetiracetam 750 mg twice daily.

Thirty-six days after hospital admission, the patient was transferred to a rehabilitation facility where she stayed for 1 month. Upon admission to the rehabilitation facility, she was unable to stand or walk on her own, had receptive aphasia, and had significant swallowing and speech impairment. She made significant improvement during her rehabilitation stay, regaining her ability to walk short distances and to communicate in simple sentences. She was advanced to a soft diet and discharged home with continued weekly rehabilitation and in-home assistance with performing daily tasks. Discharge antiepileptic medication included only levetiracetam 1,000 mg twice daily. One month after discharge from the rehabilitation facility, her PEG tube was removed. During several follow-up outpatient visits, she was noted to have ongoing upper extremity weakness and difficulty with her gait, as well as decreased coordination.

**Figure 3.**
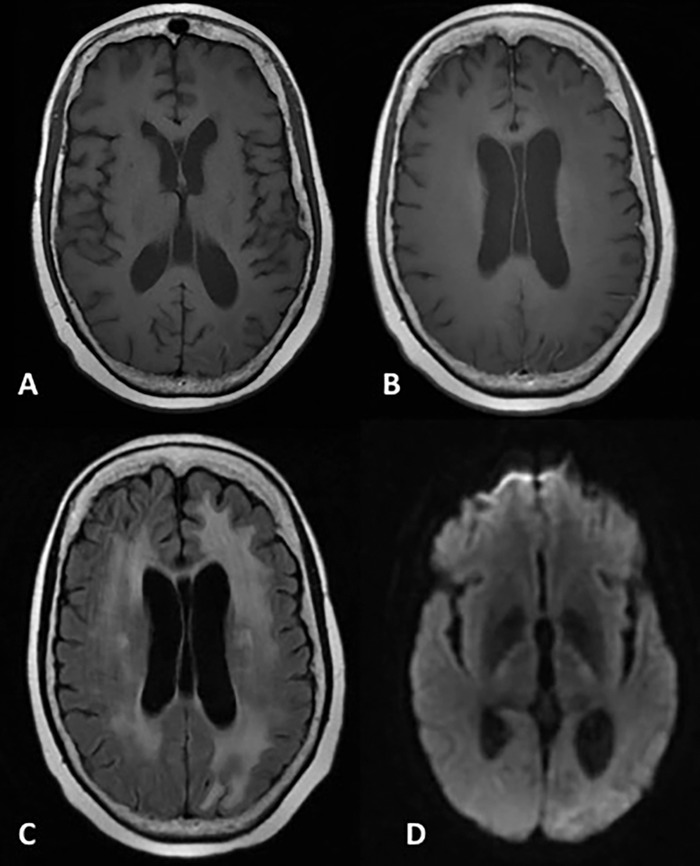
**Magnetic resonance imaging at 5-month follow-up. Axial T1-weighted images before (A) and after (B) the administration of gadolinium contrast demonstrate minimal cortical intrinsic T1 shortening along the left posterior parietal and occipital cortex consistent with laminar necrosis but complete resolution of the postcontrast cortical enhancement. T2/fluid attenuated inversion recovery image demonstrates resolution of the posterior cerebral cortical signal abnormality but new left occipital subcortical hyperintensity, indicative of gliosis and encephalomalacia (C). Diffusion weighted imaging shows resolution of diffusion restriction (D).**

On 5-month follow-up MRI, the cortical enhancement and diffusion restriction had almost completely resolved with minimal residual laminar necrosis (Figure 3).

## RADIOGRAPHIC APPEARANCE AND TREATMENT

MRI is the mainstay of radiologic diagnosis for SMART syndrome as CT typically will not illustrate any abnormalities. Characteristic MRI findings include unilateral thick gyriform cortical enhancement and corresponding T2/fluid-attenuated inversion recovery (FLAIR) hyperintensity.^1,6^ The most commonly affected areas include the occipital, parietal, and posterior frontal lobes. Subcortical and deep white matter is typically spared. Findings also do not tend to involve the deep gray nuclei or posterior fossa structures. Imaging findings are often transient and reversible. Although enhancement can last up to 12 weeks, it typically lasts 2 to 5 weeks.^1,3,4^ Diffusion restriction is often present but may be absent in the region of cortical abnormalities.^1^ In a case series by Black et al, 3 of 11 patients demonstrated diffusion restriction, but the authors hypothesized that the diffusion restriction could be attributed to superimposed infarcts.^1^ Biopsy is not typically indicated, as histopathologic findings are nonspecific and SMART syndrome is a clinicoradiologic diagnosis.^7^

There is no clear consensus regarding treatment, but therapy is usually targeted toward symptom control. In the acute setting, immediate control of seizures is important and can be accomplished with anticonvulsive therapy such as intravenous levetiracetam or fosphenytoin. Abend et al suggested that early administration of levetiracetam is effective in the treatment of status epilepticus in patients diagnosed with SMART syndrome.^8^

## DISCUSSION

SMART syndrome is a delayed complication of both whole brain irradiation and selective radiosurgery. The onset of symptoms varies widely from 1 to 37 years after initial radiation therapy, with an average 9.5 years reported by Black et al and 20 years reported by Maloney et al.^1,9^ SMART syndrome has been described in both children and adults and is typically associated with radiation doses of 50 Gy or more, although it can occur with lower doses.^10-12^ Acute symptoms consist primarily of headaches, often with a migraine-like aura. Subsequently, patients often develop confusion, seizures, and stroke-like symptoms that may include motor deficits, paresthesia, aphasia, and visual disturbances. Symptoms last from several hours to several weeks. Some patients experience full recovery, while others have recurrent transient episodes and incomplete recovery with long-term residual neurologic deficits.^9,13^

Black et al proposed a revision of the Bartleson diagnostic criteria that includes several defining features of the syndrome and MRI findings.^2^ The criteria include a remote history of external beam brain irradiation without residual or recurrent disease with symptoms that are referable to a unilateral cortical region. These manifestations include hemisensory defects, hemiparesis, aphasia, seizures, headaches, and confusion. MRI findings consist of diffuse, transient cortical enhancement that often spares the white matter and cannot be attributable to another disorder.

The pathophysiology of SMART syndrome is not well understood. Radiation is a defining precursor and a known cause of delayed neurotoxicity that can result in leukoencephalopathy and radiation necrosis.^14^ Farid et al suggested that neuronal dysfunction plays a role in SMART syndrome.^12^ Impairment of the trigeminovascular system and ion channels leads to disruption of the blood-brain barrier and subsequent lowered threshold for spreading of cortical depression, a process involved in the pathophysiology of migraine headaches. Black et al proposed that endothelial dysfunction may lead to disruption of the blood-brain barrier with subsequent marked cortical enhancement.^1^

Associated histopathologic findings include gliosis, perivascular cell infiltrates, and inflammation.^7^ Endothelial cell damage and vascular endothelial growth factor upregulation can be seen with smooth muscle proliferation and fibrinoid necrosis of the vessel wall.^15-17^ However, which, if any, of these factors is responsible for the clinical and radiologic findings is unclear, as case reports have reported no identifiable histopathologic abnormality. Shuper et al reported the case of a patient whose autopsy 1.5 years after the beginning of migraine headache episodes demonstrated no vascular damage.^6^ Black et al reported the case of a patient's brain biopsy that demonstrated nonspecific gliosis without inflammation or other histopathology related to SMART syndrome.^1^

SMART syndrome is typically reversible. In the Rigamonti et al review of the literature, 83% of patients (30/36) were reported to have had a complete recovery.^18^ Di Stefano et al reported full recovery in 85% (22/26) of patients, while 15% were left with permanent symptoms.^19^ Black et al reported that 45% (5/11) of patients in their case series had long-term symptoms, including cognitive impairment, hemiparesis, and dysphasia.^1^ Permanent imaging findings of cortical laminar necrosis were present in 27% of patients.

## DIFFERENTIAL DIAGNOSIS

The initial consideration when presented with enhancing lesions in a symptomatic patient with a history of treated malignancy is a recurrent neoplastic process. However, SMART syndrome can be distinguished by the characteristic gyriform cortical enhancement vs the solid or nodular pattern often seen in metastatic disease. CSF dissemination of malignancy can mimic the gyriform pattern of SMART syndrome, but the enhancement will be centered in the leptomeninges instead of the cortex.

Seizure-related signal changes in the setting of status epilepticus have some of the same imaging findings as SMART syndrome, including abnormal cortical and subcortical white matter T2/FLAIR hyperintensity and variable gyral or leptomeningeal enhancement.^4^ Similarly, these signal changes are not typically confined to vascular territories. In seizures, the pattern of T2/FLAIR hyperintensity often prominently involves the hippocampus, corpus callosum splenium, and thalamus, which is not typical for SMART syndrome.

Subacute infarcts often exhibit gyriform enhancement. However, findings should remain confined to a vascular territory, and the clinical scenario will differ. Our patient presented with recent-onset slurred speech and right-sided hemiparesis, initially concerning for an acute ischemic infarct. This diagnosis was ruled out when the initial MRI did not demonstrate diffusion restriction or additional findings consistent with ischemia. Additionally, in early ischemia, loss of the gray-white differentiation and edema is typical and was not demonstrated in our patient.

Infectious processes, such as encephalitis or meningitis, can have imaging characteristics similar to SMART syndrome. In meningitis, leptomeningeal T2/FLAIR hyperintensity and enhancement are often seen, as well as an exudative process in the sulci and basilar cisterns that often results in hydrocephalus. Typical imaging findings in several encephalitides are T2/FLAIR hyperintensity in the limbic system, often bilaterally. Cortical swelling with loss of gray-white differentiation can also occur, often in the medial temporal lobes and insula with sparing of the basal ganglia. Subacute hemorrhage can occur within edematous areas. These findings do not occur in SMART syndrome.

As in SMART syndrome, posterior reversible encephalopathy syndrome (PRES) demonstrates endothelial damage and vasculopathy with increased parietocortical FLAIR signal because of vasogenic edema. In PRES, however, imaging findings are typically bilateral, and enhancement is only seen in a minority of cases.

## CONCLUSION

In a patient with stroke-like symptoms, migraines, and seizures; with MRI findings of gyriform enhancement; and with a history of prior brain irradiation, SMART syndrome should be the leading diagnosis in the differential. Appropriate clinical workup is essential to exclude other potential etiologies. Familiarity with the characteristic findings of SMART syndrome and the subtle distinguishing imaging features can prevent unnecessary and invasive testing.

## Notes

This article meets the Accreditation Council for Graduate Medical Education and the American Board of Medical Specialties Maintenance of Certification competencies for Patient Care and Medical Knowledge.
